# ‘Tiny Iceland’ preparing for Ebola in a globalized world

**DOI:** 10.1080/16549716.2019.1597451

**Published:** 2019-05-07

**Authors:** Geir Gunnlaugsson, Íris Eva Hauksdóttir, Ib Christian Bygbjerg, Britt Pinkowski Tersbøl

**Affiliations:** a Faculty of Sociology, Anthropology and Folkloristics, University of Iceland, Reykjavík, Iceland; b School of Global Health, Department of Public Health, University of Copenhagen, Copenhagen, Denmark

**Keywords:** Global health, prevention and control, public policy, qualitative evaluation, emergency responders, communicable diseases, emerging, fear

## Abstract

**Background**: The Ebola epidemic in West Africa caused global fear and stirred up worldwide preparedness activities in countries sharing borders with those affected, and in geographically far-away countries such as Iceland.

**Objective**: To describe and analyse Ebola preparedness activities within the Icelandic healthcare system, and to explore the perspectives and experiences of managers and frontline health workers.

**Methods**: A qualitative case study, based on semi-structured interviews with 21 staff members in the national Ebola Treatment Team, Emergency Room at Landspitali University Hospital, and managers of the response team.

**Results**: Contextual factors such as culture and demography influenced preparedness, and contributed to the positive state of mind of participants, and ingenuity in using available resources for preparedness. While participants believed they were ready to take on the task of Ebola, they also had doubts about the chances of Ebola ever reaching Iceland. Yet, factors such as fear of Ebola and the perceived stigma associated with caring for a potentially infected Ebola patient, influenced the preparation process and resulted in plans for specific precautions by staff to secure the safety of their families. There were also concerns about the teamwork and lack of commitment by some during training. Being a ‘tiny’ nation was seen as both an asset and a weakness in the preparation process. Honest information sharing and scenario-based training contributed to increased confidence amongst participants in the response plans.

**Conclusions**: Communication and training were important for preparedness of health staff in Iceland, in order to receive, admit, and treat a patient suspected of having Ebola, while doubts prevailed on staff capacity to properly do so. For optimal preparedness, likely scenarios for future global security health threats need to be repeatedly enacted, and areas plagued by poverty and fragile healthcare systems require global support.

## Background

On 8 August 2014, the World Health Organization declared the Ebola epidemic in West Africa as a Public Health Emergency of International Concern (PHEIC) under the International Health Regulations (IHR) [1]. All three of the worst affected countries were  to address the emerging epidemic challenge without staff, stuff, space and systems [2–4]. With the epidemic seemingly out of control, and a proportionately high number of doctors, nurses, and midwives succumbing to Ebola [5], there was a growing fear of transmission beyond the region. In breach of WHO recommendations and guidelines [6], flights were cancelled and cross-border movement curtailed [7]. The epidemic caused public concern outside West Africa [8], as fear and racism found fertile ground [9–11], and in an effort to stop the international spread of the disease, all states were advised to be prepared to detect, investigate, and manage Ebola cases [1].

Preparedness as part of disaster risk reduction is defined as ‘the knowledge and capacities developed by governments, response and recovery organizations, communities and individuals to effectively anticipate, respond to, and recover from the impacts of likely, imminent or current disasters’ [12]. Yet, preparedness is also enveloped in and influenced by the socio-cultural dimension at the individual, organizational, and national levels, and measures to manage outbreaks are not always accepted or accommodated by the communities to which they are applied [13]. An analysis of eight European countries’ preparedness plans since 2009 for countering a future influenza A (H1N1) pandemic revealed that the way plans were framed varied considerably, and ‘[told] us something about how the different countries want pandemics and preparedness to be understood by the public’ [14]. More research was encouraged into cultural and social structures in the respective countries.

In Iceland, information about the Ebola epidemic in West Africa came from several sources. The Directorate of Health (DH) first reported on the epidemic on 8 April 2014 [15]. In Icelandic media, the rapid progress of the Ebola epidemic in West Africa was increasingly highlighted, and exported Ebola cases to Spain, USA, and elsewhere, were widely covered. Fear of a global epidemic was rife, and in media and online discussions, doubts were raised about the Icelandic health system´s capacity to take care of a patient with Ebola [16–18], despite its ranking as one of the best in the world [16].

On 11 August 2014, three days after WHO declared PHEIC because of Ebola, DH encouraged Icelandic citizens to avoid visits to the area, if possible, and reported that the national epidemic preparedness plan was being activated for Ebola [19]. It was elaborated by a team that involved the Chief Epidemiologist at the DH, Landspitali University Hospital (LSH), the Department of Civil Protection and Emergency Management (DCPEM), and the seven Primary Healthcare Regional Organizations in the country at the time. Key external partners were the European Centre for Disease Prevention and Control (ECDC) and WHO, in addition to Nordic collaborators in epidemic preparedness [20]. At the same time, it was regarded as highly unlikely that Ebola Virus Disease (EVD) would spread in the country [21]. Recognized scenarios included the possible appearance of an infected person in need of treatment, who could be either an Icelandic citizen who had visited or worked in one of the affected West African countries, or a person with signs of EVD on a trans-Atlantic flight in the navigation area controlled by Icelandic authorities [22–25]. On 3 November 2014, the plan was put to the test when a foreign airline made a non-scheduled landing at Keflavík International Airport due to fear of EVD in one passenger from South Africa. Parked in a closed-off area, a physician in full Personal Protective Equipment (PPE) entered the plane, but quickly ruled out Ebola [26].

Irrespective of good or bad overall performance, health systems are tested in times of crisis, such as epidemics. Here, the aim is to describe and analyse the process of establishing preparedness plans for Ebola in Iceland, with a specific focus on the perspectives and experiences of managers and frontline health workers involved in the process.

## Methods

This study is part of a larger study on the impact that the global threat of the Ebola epidemic had in Iceland [16,27]. Qualitative case study methodology was applied, perceiving the preparedness planning and training process as the case with clear boundaries of the initiation, process, and wrap-up of preparedness planning and training. The study was conducted in April-May 2016, and the interviewed participants were administrators and frontline health professionals central to the case, so as to explore their perspectives and experiences concerning Ebola preparedness [28,29]. Staff in managerial positions were contacted by one of the authors (GG) for permission to interview them based on their role in the preparedness plan. To identify potential interviewees in the Ebola Treatment Team (ETT), the director of the team listed relevant email contacts. Those who responded positively were subsequently invited for an interview, conducted in Icelandic by one of the authors (ÍEH), a physiotherapist. In case interviewees suggested other potential participants, they were invited through email to participate. A similar methodology was applied to identify participants from the Emergency Room (ER). They were included in order to represent frontline health workers who worked in the only ER in Reykjavík, where persons exposed to EVD were most likely to first seek care in case of acute illness.

Three separate interview guides were developed – one each for managers, ETT, and ER respectively (see supplementary material). The interviews included open questions probing the role of their institution in preparedness, the experience of the training process, challenges encountered or expected, and any dilemmas that they may have experienced in relation to the preparedness plan. The recruitment of participants was concluded when saturation was reached. Each interview was recorded and took about 20 to 60 minutes; they were then transcribed and analysed using thematic analysis. The data material was read through repeatedly, sorted, and categorized, based on the participants’ priorities in the representation of their views. From this exercise, three broad themes were inductively identified that corresponded to critical perspectives introduced by the participants.

Permission to conduct the study was granted by Iceland’s National Bioethics Committee (VSN-15–192) and Landspitali University Hospital (LSH 13–16, 4 February 2016). Reporting on the results was guided by the COREC guidelines [30]; however, to ensure anonymity of the respondents within the small community of staff who took part in the preparedness activities, participant information is not associated to quotations.

## Results

### Theme 1 – getting the job done

The Icelandic Ebola Preparedness Plan included the establishment of an ETT within LSH [31], and the preparatory activities engaged more than two hundred staff across all of its departments. The ETT consisted of about 50 healthcare professionals who had volunteered to participate, including 11 doctors and 28 nurses, a few laboratory technicians, radiologists, and auxiliary nurses. They attended special training sessions focused on protocols for admission and treatment of a patient with EVD, the donning/doffing of PPE, and personal protective measures during patient care. A new provisory unit was designed to be set up on the ground floor to minimize the risk of infection spreading to other units within the hospital, with two rooms specifically identified for the care of a patient with EVD [31].

Managers’ accounts of this period elaborated the complexity of preparedness planning in terms of the involved institutions, actors, procedures and requirement of the plan. One manager concluded:

You get no discount. You can never go the shorter way. There was always something that surprised you. We thought this was a lot like a three headed monster, so when you chopped off one of its heads, three other emerged, every solution was followed by more problems.

The health professionals who volunteered to join ETT did so for different reasons. Ebola preparedness was ‘a job that had to be done’, and ‘someone had to do it’. Some referred to ethical or professional obligations:

This is just a part of being a nurse, to encounter situations that can be dangerous to you or someone else, but you have made this decision and you deal with it.

Some connected their decision to their ‘action gene’ or ‘addiction to taking risks’, while others said they had already raised their kids and had years of experience, including work with other epidemics, such as HIV. Yet, the practice of volunteering in the preparation was questioned. One participant said:

We learned that we could not rely on volunteers … when you work in an infectious disease department you cannot choose what infections you want to work with.

ER staff indicated that for them working in the ER was enough of a risk to take, no reason to expose oneself even more by joining the ETT, and appreciated that others had volunteered.

All participants noted that co-operation and communication had generally functioned well during the preparedness planning, with information flowing both ways. Short communication lines within the healthcare system were perceived as both a strength and a weakness; a strength, insofar as people knew each other, but a weakness because of the uneven burden of workload. Staff of the ETT and in the ER felt they had been well-informed, and that openness and honesty had characterized the planning and diminished their initial fear. Those in managerial positions had listened and taken their opinions into consideration. One said:

They were honest, no one was hiding anything, everything was on the table, no one tried to make things more appealing and say that everything would be OK, they just told us about things as they were.

### Theme 2 – trust, doubt and fear

Both management and participants from the ETT and ER expressed their ambiguity in terms of trust, doubt, and fear. Participants conveyed trust in the health system and their own role as health professionals, while at the same time admitting to facing formidable challenges during the elaboration of the preparedness plan. Facilities for isolation and treatment of patients with Ebola were less than perfect:

We assessed how we could use the department … and change it in just a few hours into some kind of an isolation unit that we could possibly use.

Some compared this short-term isolation facility to a ‘camping site’, as the facilities were too provisional and not comparable to those found elsewhere. There was also doubt about how many Ebola patients LSH would be able to care for: ‘Maybe one or two patients, barely more’.

Respondents believed that the training and education of the members of the ETT and ER had been satisfactory. They felt that it had been proportionate to the risk, while some were concerned about the lack of staff. Nonetheless, there were contradictions on the division of labour among the professionals, exemplified by different ideas on how to proceed if a patient suspected of having an EVD came in an ambulance to the LSH for treatment. Almost all participants stated that they were ready to do their part in the Ebola response, or ‘as ready as [we] could be’.

There were diverse opinions on what it meant to be ready: to treat one confirmed case of Ebola, one suspected case, or more EVD patients? When asked if Ebola was a real threat to the country, participants usually referred to how easy it was to travel the globe: ‘Yeah, why not, the world is getting smaller’. Although Ebola was thought of as a real danger by many, some participants expressed difficulty in taking their training seriously, doubting that Ebola would ever reach Iceland. One respondent said:

People were dedicated in the beginning, but when the news appeared that Ebola was receding, that diminished, and I never felt like this formally ended.

Participants described their relief that nothing really happened, while emphasizing the need to experience a real situation to evaluate the preparedness efforts. One participant said that ‘a little bit more seriousness [would have been] needed in the PPE practices’.

It was taken as a manifestation of fear that some of the staff in the communicable disease department of the LSH refused to take part in the ETT. When describing their fears, ETT members frequently connected it to their working conditions. Many of them were afraid that they would not get the best PPE, others that they would not do the donning/doffing correctly and, lastly, they were worried about work performance while in the PPE. One participant said:

What bothered most of us was how uncomfortable the PPE was and I think that made people nervous: “How will I manage working in this for hours?”

Another described the donning/doffing process like a ‘complicated ballroom dance’. Moreover, participants were afraid of ‘unknown territories’, that is, they did not know the hospital ward, they were supposed to work in, and some team members had no recent experience of clinical work. One participant said:

I didn’t think these [non-clinical] people belonged in the team, because this is a very clinical environment in addition to having to be in this costume [PPE] with the risk of becoming infected by mistake.

Those with non-clinical background were, however, aware of their limitations:

I realized that I would not be the one in the front, I would not be managing patients directly.

The importance ascribed to teamwork was evident in relation to fear. Participants described fear of working with people they had not worked with before:

The weakest link in the preparation was that even though I knew their faces, I had never worked with them.

Another issue was no-show by some team members in training sessions or in lectures:

This is team-work, one does this and the other one does this, [we] help each other. Then you don’t want to be working with someone who didn’t show up.

Another one said:

There were a lot of doctors who just dropped in, dropped out, and then dropped in again. I asked myself: Are these individuals … ready to take this on?

Participants in the ETT mentioned the precautions they took or intended to take to cope with their feelings of fear, should Ebola emerge in Iceland. A major precaution was planning to avoid contact with the family while working with Ebola patients. One participant said: ‘You thought … about your children at school … parents in the neighbourhood …’ if they knew (s)he was working with an Ebola patient. For them, it was important they would have access to special accommodation in case of clinical EVD work ‘so I wouldn’t be exposing anyone or creating hysteria’. ETT members mentioned the extra insurance offered as a prerequisite for taking part in the team. ‘The normal insurance for LHS staff would not cover everything if we were to become sick or even lose our lives.’ Amongst ER staff, the matter of insurance did seem to be less of an issue compared to the ETT. One respondent said: ‘You are used to being at risk by many disease threats’. Furthermore, the issue of higher salaries and risk commission came up in the interviews, but overall did not matter as much to the participants as the insurance, or assurance of accommodation in case of need.

### Theme 3 – the Icelandic way

Characteristics associated with Iceland and the Icelandic people were referred to repeatedly by participants. The concept ‘Tiny Iceland’ was often mentioned and emerged with positive and negative connotations. ‘Tiny Iceland’ referred to the size of the country and population and its perceived capability to still ‘get the job done’. even though compromises had to be made. Comparing how Iceland handled its responsibilities differently from other countries of a larger size was often brought up, both with pride in Iceland as a strong independent nation, and with insecurities about its capacity in comparison to other countries. It was pointed out that since the preparedness process was in the hands of a few people, everyone knew their role. As one administrator said:

This little hospital system, as complicated as it might seem every day, gives you the chance to just pick up the phone and call the one in charge.

Being a small population presents challenges regarding resources, infrastructure, and specialized medical training to comply with standards of international actors. Notions of Icelanders as resilient in spite of shortcomings were common; referring to the experience of preparedness planning and training, one health staff said:

It was very much the Icelandic way, we’ll manage, we’ll work it out, and there was so much ingenuity.

This notion of a particular Icelandic approach to coping, in spite of shortcomings, was also detected more generally, as in the statement:

Would it have worked? Yes, it would have worked. Would it have been optimal? We cannot say, it would have been optimal; we can say, it would have been sufficient.

In contrast to this, there were concerns about whether Icelandic aid workers falling ill in Ebola-affected countries should be transferred to Iceland or to hospitals in other Nordic countries with better isolation units. Some of the participants trusted that patients with EVD would not be transferred to Iceland. One participant stated:

You heard that Norwegians were criticized for transferring their aid worker from Africa to Norway. We don’t know what would have happened if they would have transferred an Icelander into the country.

Another participant said:

We don’t have good enough isolation units – you are not supposed to send patients to a hospital that is less than 100%. I thought there was assurance in that.

## Discussion

During the devastating Ebola epidemic in West Africa that spread to neighbouring sub-Saharan countries, North America, and Europe [32], preparedness plans were widely elaborated and later evaluated. Evaluations have, for example, been conducted in 11 African countries close to the epidemic [33], in the EU region [34,35], and the US [36]. Here we present data from a qualitative case study on the process, and experiences with establishing a preparedness plan for Ebola in Iceland in 2014. Interviews with staff who were engaged, either as administrators or frontline healthcare workers, alert us to the manner in which geographic, demographic, cultural, and organizational characteristics shaped the response. The results show that the process of establishing and training for preparedness was permeated by ambiguities of pride and pragmatism, trust, doubts, and fear.

‘Getting the job done’ (theme 1) refers to the multitude of tasks and considerations that surrounds and feeds into the preparedness plan itself and are necessary for successful planning and implementation. Using the metaphors of ‘hard core’ and ‘soft periphery’, Langley and Denis [37] emphasize the importance of relatively ‘peripheral’ concerns and processes for planning and implementation of new interventions. The hard core represents the actual intervention or goal, e.g. implementation of a preparedness plan. The soft periphery refers to all the contextually important networking, negotiations, and agreements necessary to deliver the hard core. If the soft periphery is neglected, it will cause multiple challenges in the implementation process, and the beneﬁt of the hard core, the intervention itself, may not transpire as anticipated. Due attention to the soft periphery may, however, considerably promote the delivery of an innovation, and secure support from important stakeholders. In our data, one manager speaks of the preparedness process as dealing with a three-headed monster where every solution was followed by new problems. The data indicate that the process of dealing with ‘the three headed monster’ was given due attention as a means to successfully develop Iceland’s preparedness plan. Comprehensive consultations and the involvement of many associated institutions were mentioned. Still ambiguity remained with some staff in terms of division of responsibilities and tasks – e.g. when transporting a patient potentially infected with Ebola from the airport to the hospital, and other such activities.

During epidemics, rumours, gossip, and unreliable information on the news and social media spread rapidly, resulting in so-called ‘infodemics’ [38]. The West African Ebola epidemic was covered widely by media [39], and the fear of Ebola reached every corner of the world, exemplified by travel bans from affected countries, and trade barriers [40], in contrast to the ongoing epidemic in the Democratic Republic of Congo [41,42]. In our second theme, trust, doubt, and fear of health workers were represented. Although all intentions were good, concerns remained about the suitability and safety of the isolation ward, the PPE, and other tools, as well as adequate engagement of colleagues who might potentially work alongside them, in case an Ebola patient came to Iceland. The foreignness of putting on, removing, and working from within a PPE and the trustworthiness of available PPE were mentioned. In preparedness efforts in other countries, scarcity of resources in relation to manpower demand and problems with training and protocols involving PPE were common challenges [35]. Similar problems were encountered in Iceland. Provisory treatment facility had to be designed, called ‘camping site’ by some, in contrast to facilities found elsewhere [43]. Further, the ETT was established based on voluntary recruitment rather than on the staff’s assigned roles within the healthcare system, a procedure that was deemed less than optimal. The members of the ETT pointed out that they had never worked together as a team under circumstances that demanded strict adherence to infectious control procedures. This eroded trust, compounded by the *laissez-faire* attitude of some of its members during the preparation exercises, possibly due to other competing tasks in a busy hospital and insufficient resources that hampered full participation [44]. Further, it was a constraint that simulation exercises were not an option, found to be an important element in preparation for epidemics [35]. This might have resulted in less than optimal staff protection for those who would have been in direct contact with an infected patient, as reported during the SARS epidemic in Canada [45,46].

Anthropological work on emergency preparedness emphasizes the connectedness between health professionals, technological devices, and knowledge as a pre-requisite for successful preparedness. Wolf and Hall present preparedness efforts as a form of governance that involves human bodies (those of health professionals), clinical architectures (e.g. isolation wards), and technical artefacts (gloves, protective suits, disinfectants, etc.) [47]. During preparedness training and implementation, ‘nursing bodies are transformed into instruments of preparedness’, and become part of infrastructural arrangements. Health professionals are, here, both vulnerable and powerful tools in the management of contamination. The authors argue that successful planning, training, and implementation of a preparedness plan require such intrinsic connectedness. In the case of Ebola preparedness in Iceland, health professionals draw our attention to dilemmas of connectedness, and their assessment of the fact that these shortcomings might hamper the mobilization of ‘preparedness within the human body’ – that is, the embodied experience, routine, and tacit knowledge which Wolf and Hall state are key to successful implementation. Repeated enactment of receiving and treating a patient with Ebola within experienced and trustful teams would probably enhance such embodiment, provided that there is justified trust in the involved technology. In addition, repetition would also strengthen the ‘soft periphery’ of preparedness, and divisions of responsibilities would be clearer manifested.

In the third theme, we observe how notions of the ‘Icelandic way’ help participants make sense of ambiguities about Ebola preparedness. Loftsdóttir explored how people negotiated the imagination of the local and the global during the 2008 economic crisis in Iceland [48]. Notions of the intrinsic character of Iceland, and of being Icelandic, serve to underscore certain points and explain positive and negative experiences with the preparedness plan. Iceland is far away from the continents, but still connected through global needs for policy, risk of contamination, and dependency in terms of collaboration, in emergencies emerging from elsewhere. In our study, participants highlighted the importance of believing in oneself and the ‘Icelandic way of doing things,’ summed up in the paraphrase ‘þetta reddast’ (things always have a way of working out in the end). The preparedness plan had to be completed, and adapted to Iceland’s particular global situation.

In the 21st century, the world has faced new epidemic threats, such as SARS, and old scourges such as the plague have resurfaced [38]. One of the main findings on Ebola preparedness measures in the EU was that measures taken were based on past preparedness and experience of other epidemics, such as SARS and H1N1 [35]. Further, key stakeholders within each country found their measures to have been adequate for dealing with a single case of Ebola, as was the case in Iceland. A preparedness plan for pandemic influenzae in Iceland was elaborated in 2006 – activated in response to the H1N1 epidemic in 2009 – and revised in 2016 [49]. During the elaboration of these plans, communication among the different levels of the healthcare system and supporting agencies, such as the DCPEM, had been clearly defined, and proved to be useful in the preparedness for Ebola. Further, as found important in preparedness activities for pandemic influenzae elsewhere [44], honesty, transparency in communication, and sharing of information from managers to front-line health professionals, was found to be critical. It gave a feeling of being involved, and mitigated the fear that is so frequently encountered during epidemics [38].

## Conclusions

Iceland was far away from the epicentre of the Ebola epidemic in West Africa. Yet this case study shows that health professionals felt the strain of possibly having to treat one or more patients with EVD. Their situation stands in sharp contrast to the situation in the three worst affected West African countries that lacked staff, stuff, space, and systems to effectively address the challenge of EVD. Although Icelandic health professionals had trust in the national healthcare system, and in their own capacity, doubt and fear influenced the reflections on preparedness planning of both administrators and healthcare staff. References to national identity and the characteristic of an ‘Icelandic approach’ to handling challenges assisted participants in coming to terms with the experienced shortcomings of the preparedness plan, and underscored the pride in the ingenuity applied in the process. These references negotiate the role and character of the nation of Iceland, and its role in a globalized world, as both a small and isolated nation on one hand, and a central and capable one, on the other.

The experienced ambiguity needs attention in a health system and among healthcare staff that have to act resolutely and unfailingly, should they be placed in charge of containing contamination. This study points to the necessity of repeatedly re-enacting, as realistically as possible, the likely scenarios of receiving and treating one or more patients infected with Ebola (or other contagious global health threats) as a routine matter. This would assist in the identification of overlooked ‘soft periphery’ concerns, and promote embodied preparedness among teams of health care staff on the frontline.

## Paper context

The manuscript builds on the work of Íris Eva Hauksdóttir towards a MSc in Global Health, Section of Global Health, Department of Public Health, Copenhagen University, Denmark.
