# Supplementary material for: ‘Tiny Iceland’ preparing for Ebola in a globalized world
Source: Glob Health Action. 2019 May 7;12(1):1597451. doi: 10.1080/16549716.2019.1597451 (PMC6507955; doi:10.1080/16549716.2019.1597451)
Supplement: Supplemental Material [file ZGHA_A_1597451_SM5221.docx]

**Interview guides**

1. **Planners of the response plan**

**Introduction**

Thank you so much for coming. As you have read in the introduction letter, my name is Iris and I am a master student in Global health at University of Copenhagen. At the moment I am doing my master’s thesis, which is about preparedness for Ebola in Iceland. To get a better picture of the preparedness, I will be taking interviews with key informants, taking part in the preparedness measures, both planners and partakers, such as front line health care staff.

**Overall study objective:**

The main objective of the study is to explore the institutional partners and their roles in preparedness for Ebola in Iceland and the perspectives of frontline health worker’s on the preparatory process and their role in Ebola response.

Before we start would you agree to sign this concept form?

If you have any questions through the interview, feel free to ask.

**Overall theme of the interview**

Overview of the preparedness measures in Iceland

**Structural questions and grand tour**

Can you start by telling me a little bit about yourself

Name

Age

What your job involves

How are you connected with Ebola preparedness in Iceland

Probe…

**Themes**

Institutions role in preparedness

Can you tell me about you institution’s part in the preparedness measures?

How was this experience?

Inititation of the preparedness

Can you tell me about the initiation of the preparedness measures?

How did it start?

Who were involved in the initiation?

Structure of an institution’s preparedness plan

Who were the main participants and stakeholders in the planning?

Did you co-operate with other institutions?

What contributed to shaping the preparedness plans?

Probe for more. Such as guidelines or former preparedness plans

Main emphasis of the preparedness plan

Can you tell me what the preparedness plan involves, what factors were included in the plan?

Points of entry?

Community (primary respondents: emergency departments, primary care, public health)?

Inland transportation (ambulance services)?

Treatment in designated hospital?

Medical evacuation?

Frontline health worker’s participation (Ebola team members or other frontline health care workers involved in the plan at each institution)

Recruitment, how did it go?

Who were appointed to the team

What occupations and why those occupations?

What kind of people?

Something that characterizes the group

Training and education of health professionals

Main emphasis in the training

How did it go?

Communication with others

How was the communication with other institutions involved?

Directorate of health

Ebola team

Other ports of entry

Partners abroad

Was the response plan tested at some point?

How did it go?

Readiness for Ebola

What challenges do you foresee in terms of the institutions part in the preparedness plan?

Strengths identified?

Vulnerabilities identified?

Gains from making the preparedness plan

Do you feel like your institution gained something from this experience in planning and training for Ebola?

Staff skills?

Organizational preparedness?

Physical infrastructure?

End of the interview

Do you have any other comment concerning the preparedness plan?

Summmarize and ask if I have understood the information given from the informant correctly

Thank the informant for participating

II. Ebola Treatment Team (ETT) members

**Introduction**

Thank you so much for coming. As you have read in the introduction letter, my name is Iris and I am a M.Sc. student in Global Health at University of Copenhagen. At the moment I am doing my master’s thesis, which is about preparedness for Ebola in Iceland. To get a better picture of the preparedness, I will be taking interviews with key informants, taking part in the preparedness measures, both planners and partakers, such as frontline health care staff.

**Overall study objective:**

The main objective of this study is to explore and evaluate the establishment Ebola preparedness in Iceland and how this preparedness and distribution of roles are perceived by different authorities and frontline health workers

Before we start would you agree to sign this concept form?

If you have any questions through the interview, feel free to ask.

**Main theme of the interview**

Role in preparedness and perceptions about ebola and the preparedness itself.

**Structural questions and grand tour**

Can you start by telling me a little bit about yourself

- Name
- Age
- What your job involves
- How you are connected with Ebola preparedness in Iceland

**Themes**

Role in Ebola preparedness

- Can you tell me about your role in the Ebola preparedness measures in Iceland?
  - How do you perceive this experience?
- How did you end up in the Ebola team?
  - Were you offered some compensation for being in the team?

Reasons for joining the Ebola team-risk taking

- - What were you reasons for joining the team?
    - Probe for reasons
- What considerations did you have about joining the team

Probe- what did you discuss with your family?

- Did you have any doubts when joining the team?
  - What about the other people on the team?

Training and education:

- What did the training involve?
  - Probe for more
    - Simulation exercises?
    - Face to face training?
    - Online training?
    - Other?
- Did the training go on regularly through the whole period?
- Your thoughts about the training
  - Positive and negative feedback

Communication

- Do you believe you were informed well enough on the Ebola epidemic and the relevant procedures and guidelines?
  - Probe for more
    - Confusion?
    - Too much, too little?
- Were you updated regularly?
  - Where did you get the updates from?

Co-operation

- With other members of the team?
- Primary health care facilities?
- Other health care staff at the hospital
- Were you respected by the planners of the preparedness plan?
  - Do you feel like they took your thoughts and perspectives into consideration?
  - Did you have a say in the planning of your response?

Preparedness:

- What challenges do you foresee in terms of your institutions part in the preparedness plan?
  - Probe for strengths and weaknesses identified in the preparedness measures
    - Probe-how did you come to this conclusion?
- Do you feel prepared for taking on an Ebola case?
  - If yes, why?
  - If no, why?
- Personal protective equipment?

Perceptions of Ebola

- Do you believe Ebola was a real threat to Iceland? To the world?

Stigma, fear, worries:

- Family, friends
- Your reputation
- -What do you think of the health care workers who went abroad to take part in the epidemic?
- Worries of insurance

End of the interview

- Do you have any other comment concerning the preparedness plan?
- Summarize and ask if I have understood the information given from the informant correctly
- Thank the informant for participatin

III. Interview guide for frontline health workers - working at LSH’s ER

**Introduction**

Thank you so much for coming. As you have read in the introduction letter, my name is Iris and I am a M.Sc. student in Global Health at University of Copenhagen. At the moment I am doing my masters thesis, which is about preparedness for Ebola in Iceland. To get a better picture of the preparedness, I will be taking interviews with key informants, taking part in the preparedness measures, both planners and partakers, such as frontline health care staff.

**Overall study objective:**

The main objective of this study is to explore and evaluate the establishment Ebola preparedness in Iceland and how this preparedness and distribution of roles are perceived by different authorities and frontline health workers

Before we start would you agree to sign this concept form?

If you have any questions through the interview, feel free to ask.

**Main theme of the interview**

Role in preparedness and perceptions about Ebola and the preparedness itself

**Structural questions and grand tour**

Can you start by telling me a little bit about yourself ?

- Name
- Age
- What your job involves
- How you are connected with Ebola preparedness measures in Iceland

**Themes**

Role in Ebola preparedness

- Can you tell me about your role in the Ebola preparedness measures in Iceland?
  - How do you perceive this experience?

Training and education:

- What did the training involve?
  - Simulation exercises?
    - How does this takes place, can you give an example
  - Face to face training,
    - How does this takes place, can you give an example
  - Online training
  - Other
- Did the training go on regularly through the whole period?
- Your thoughts about the training
  - Positive and negative feedback

Communication

- Do you believe you were informed well enough on the Ebola epidemic?
- You had access to relevant procedures and guidelines?
  - Probe for more
    - Confusion?
    - Too much, too little?
- Were you updated regularly?
  - Where did you get the updates from?

Co-operation with

- Primary health care facilities?
- EMTs
- Other health care staff at the hospital
  - Ebola team?

Preparedness:

- What challenges do you foresee in terms of your institution’s part in the preparedness plan?
  - - Probe-how did you come to this conclusion?
- Do you feel prepared for detecting an Ebola case?
  - If yes, what is it that makes you feel prepared?
  - If no, what is it that makes you not feel prepared?
- Personal protective equipment?

Stigma, fear, worries:

- Family, friends
- Your reputation
- What do you think of the health care workers who went abroad to take part in the epidemic?
- Worries ((of insurance?

Perceptions on Ebola

- Do you believe Ebola was a real threat to Iceland? To the world?
  - Probe for reasons
- Did you consider joining the Ebola team?
  - If yes, for what reasons?
  - If no, for what reasons?

End of the interview

- Do you have any other comment concerning the preparedness plan?
- Summarize and ask if I have understood the information given from the informant correctly
- Thank the informant for participating
